# α6-Integrin alternative splicing: distinct cytoplasmic variants in stem cell fate specification and niche interaction

**DOI:** 10.1186/s13287-018-0868-3

**Published:** 2018-05-02

**Authors:** Zijing Zhou, Jing Qu, Li He, Hong Peng, Ping Chen, Yong Zhou

**Affiliations:** 1 Department of Medicine, Division of Pulmonary, Allergy and Critical Care Medicine, University of Alabama at Birmingham, Tinsley Harrison Tower 437B, 1900 University Blvd, Birmingham, AL 35294 USA; 2 Department of Respiratory Medicine, The Second Xiangya Hospital, Central-South University, Changsha, 410011 Hunan China

**Keywords:** α6-Integrin, Stem cell; Stemness, Niche, Alternative splicing

## Abstract

α6-Integrin subunit (also known as CD49f) is a stemness signature that has been found on the plasma membrane of more than 30 stem cell populations. A growing body of studies have focused on the critical role of α6-containing integrins (α6β1 and α6β4) in the regulation of stem cell properties, lineage-specific differentiation, and niche interaction. α6-Integrin subunit can be alternatively spliced at the post-transcriptional level, giving rise to divergent isoforms which differ in the cytoplasmic and/or extracellular domains. The cytoplasmic domain of integrins is an important functional part of integrin-mediated signals. Structural changes in the cytoplasmic domain of α6 provide an efficient means for the regulation of stem cell responses to biochemical stimuli and/or biophysical cues in the stem cell niche, thus impacting stem cell fate determination. In this review, we summarize the current knowledge on the structural variants of the α6-integrin subunit and spatiotemporal expression of α6 cytoplasmic variants in embryonic and adult stem/progenitor cells. We highlight the roles of α6 cytoplasmic variants in stem cell fate decision and niche interaction, and discuss the potential mechanisms involved. Understanding of the distinct functions of α6 splicing variants in stem cell biology may inform the rational design of novel stem cell-based therapies for a range of human diseases.

## Background

Integrins are transmembrane glycoproteins composed of an α and a β subunit which are linked via noncovalent bonds. Most integrin subunits including α6 (also known as CD49f) contain a short cytoplasmic domain. The α6 subunit associates with the β1 or β4 subunit to form α6β1 and α6β4 integrin heterodimers. α6β1 is expressed on a variety of cell types and functions as a cellular receptor for matrix laminin [1]. α6β4 is found on the basal surface of polarized epithelial cells where it is located at the hemidesmosome adhesion complex [2]. In cells expressing both β1 and β4, α6 appears to preferentially bind to β4 [3]. Two unique regions (554–561 amino acids and 641–690 amino acids) in the extracellular domain of α6 are likely responsible for this preferential association [4]. To date, over 30 different stem cell populations have been found to express α6 integrins on the plasma membrane [5]. Transcriptional profiling analyses from independent groups have identified *ITGA6*, the gene encoding α6 subunit, as a signature gene of embryonic stem cells (ESCs), neural stem cells (NSCs), and hematopoietic stem cells (HSCs) [6–8]. In addition to acting as a “stemness” biomarker, there is increasing evidence that α6 integrins (α6β1 and α6β4) confer the functional characteristics of stem cells. Alternative splicing of precursor mRNA occurs with many integrin subunits, including α6 [4]. Alternative splicing increases the complexity of gene expression and is thought as a strategy for modifications of the function of encoded gene products. In this review, we summarize the current knowledge regarding the structural variants of the α6-integrin subunit. We highlight the spatiotemporal expression of α6 cytoplasmic variants in embryonic and adult stem/progenitor cells, and discuss the potential roles of α6 cytoplasmic variants in stem cell fate determination and niche interaction.

### Structural variants of α6-integrin subunit

**Fig. 1:**
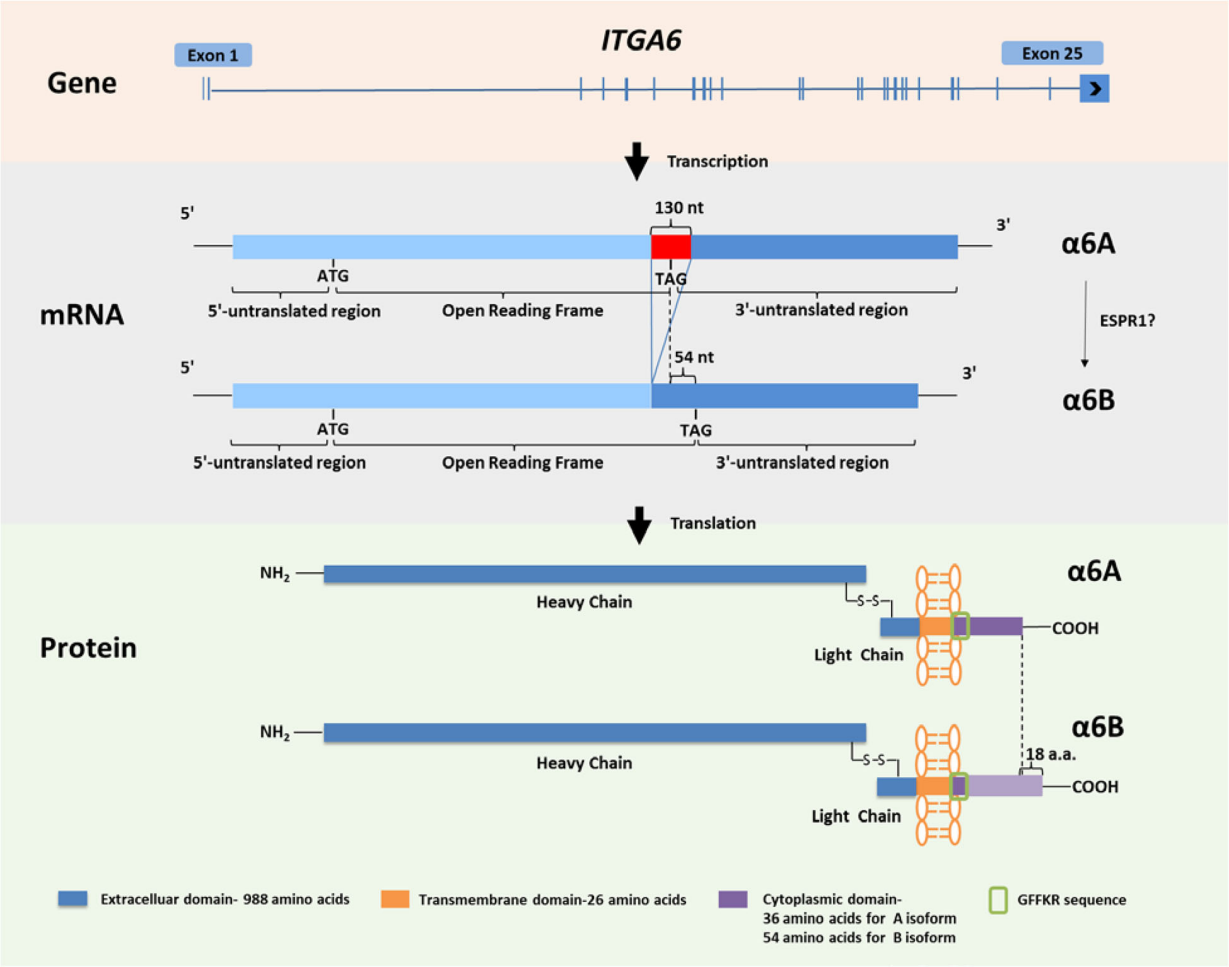
Schematic depiction of *ITGA6* gene and mRNA and protein of two identified α6 cytoplasmic variants. Human *ITGA6* gene contains 25 exons and is transcribed into prototypic α6A pre-mRNA. Alternative splicing of α6A pre-mRNA at exon 25 deletes 130 nucleotides (nt) containing the original stop codon. This deletion results in a frameshift of the downstream coding sequences and generation of a new stop codon 54 nt downstream of the original stop codon. The messenger RNAs of α6A and α6B are translated into two transmembrane protein isoforms, in which α6B isoform is 18 amino acids (amino acids) longer than and bears a poor homology with the α6A isoform

The full-length of the human prototypic α6 transcript (α6A) consists of a 5′-untranslated region (146 nucleotides), an open reading frame (ORF; 3219 nucleotides), and a 3′-untranslated region (2264 nucleotides) (Fig. 1). The ORF encodes a putative signal peptide (23 amino acids), an extracellular domain (988 amino acids), a transmembrane region (26 amino acids), and a short cytoplasmic domain (36 amino acids) [4]. A major alternative splicing of α6 occurs in the coding region of the cytoplasmic domain. Deletion of 130 nucleotides in this region results in a frameshift that eliminates the original stop codon, generating an alternative splicing variant (α6B) that is 18 amino acids longer than α6A. This also results in a high number of charged amino acids (24 out of 54) in the α6B isoform [9]. α6A and α6B bear no sequence homology at the cytoplasmic domain, except a GFFKR sequence common to all α-integrin subunits at the N-terminus of the cytoplasmic domain [10] (Fig. 1). Epithelial splicing regulatory protein 1 (ESPR1) is known to promote exon skipping by binding to the consensus UGG-rich motif in either introns or exons. Mutations of the UGG motifs downstream of exon 25 in *ITGA6* abolishes ESRP1 binding to and ESRP1-dependent exon inclusion of *ITGA6* [11]. Furthermore, loss of ESRP1-mediated mRNA splicing results in deletion of exon 25 from the mature mRNA and generation of α6B with an alternative cytoplasmic domain [12]. These findings suggest that ESPR1 is associated with the generation of α6 cytoplasmic variants. Regarding the nomenclature of α6 cytoplasmic variants, it should be noted that the prototypic α6A is designated as integrin alpha-6 isoform B preproprotein (NP_000201) and alternative splicing variant α6B as integrin alpha-6 isoform A preproprotein (NP_001073286) in the National Center for Biotechnology Information (NCBI) database.

In addition to the cytoplasmic variants, it has been reported that human *ITGA6* contains alternative X1 and X2 exons [13]. Alternative splicing of exon X2 yields two extracellular domain variants, α6X1 and α6X1X2 [14]. α6X1 expression is relatively ubiquitous, whereas α6X1X2 expression is restricted to certain types of tissues and cell lines. α6X1 and α6X1X2 do not appear to differ in ligand specificity and affinity [13]. The functional role of α6 extracellular splice variants remains to be determined. Furthermore, a smaller form (70 kDa) of the α6 variant, termed α6p, has been identified in human prostate, colon, and epithelial cancer cell lines [15]. α6p corresponds exactly to the ORF encoded by exons 13–25 of α6A. It contains the “stalk region” of the extracellular domain, the transmembrane region, and the cytoplasmic domain of α6A. Rather than alternative splicing of precursor mRNA, α6p results from urokinase-type plasminogen activator (uPA)-mediated proteolytic cleavage of the extracelluar domain of α6A after it is presented on the cell surface [16]. Because of the absence of the entire β-propeller domain, α6p is believed to function as an inactive receptor for cell adhesion to the extracellular ligand [15]. Additionally, the amino terminal fragments shed from α6A may have a functional role as well.

α6 mRNA is translated into a single protein precursor which further undergoes furin endoprotease-mediated cleavage in the extracellular domain [17]. The cleavage yields a heavy chain (110 kDa) and a light chain (30 kDa) that are noncovalently linked by disulfide bonds (Fig. 1). However, an uncleaved form of α6 has been reported in differentiating lens fiber cells [18]. The heavy chain of α6 contains most of the extracellular domain, whereas the light chain contains the cytoplasmic domain, the transmembrane domain, and the remaining extracellular domain [9]. The endoproteolytic cleavage of α6 may provide a conformational flexibility for α6 to bind the ligands [19].

### Spatiotemporal expression of α6 cytoplasmic variants in embryonic and adult stem/progenitor cells

The cytoplasmic variants of α6A and α6B are differentially expressed in developing mouse embryos. α6B(β1) expression is present at all embryo stages and is more widespread than α6A(β1) expression [20]. α6B is the only splice variant found in the developing nephrogenic system and the central and peripheral nervous systems [20], suggesting that α6B(β1) may play an important role in the development of nephrogenic and nervous systems. In contrast, α6A(β1) is expressed much later than α6B(β1), beginning in 8.5–9.5 days post-coitum embryos, and its expression is restricted to a few organs, including the developing heart, epidermis, and dental primordia [20]. Since α6 is the only known α subunit that associates with β4, areas where both α6 and β4 proteins are present presumably represent the presence of α6β4 integrins. It was found that β4 protein was absent in early post-implantation stages, but was present in the epidermis and digestive tract of embryos 12.5 days post-coitum [20], suggesting a functional role of α6Aβ4 in the development of epidermis and epithelium of the intestinal tract.

In the early post-implantation embryos, heart is the major site where α6A(β1) expression was observed [21]. Quantitative confocal microscopy shows that α6A expression is increased from the outer to the inner layers of the myocardium. Substitution of α6A by α6B in mice does not impair the development and function of the heart [22], suggesting that α6A(β1) is not essential for the differentiation of cardiac muscles.

It has been observed that isoform switching of the predominant α6 from α6B to α6A occurs during lens cell differentiation in both chicken and rat embryos [23, 24]. In undifferentiated central lens epithelial cells, α6B is most highly expressed in the equatorial epithelium, and expression of α6B begins to drop as cells initiate their differentiation. In the cortical fiber zone where lens differentiation occurs, α6A expression is high and predominates until cortical fiber cells became terminally differentiated [23, 24]. The isoform switching from α6B to α6A predominance has been confirmed in vitro in a FGF-induced rat lens fiber cell differentiation model [24]. Immunoprecipitation of biotinylated microdissected fractions of chick embryo lens has demonstrated that both β1 and β4 are expressed by the E10 chick embryo lens [23]. β1 is strongly expressed in the germinative and transitional zones of rat lens, where cells proliferate and differentiate, respectively, suggesting that β1 coupling with α6, presumably α6A, may play a role during rat lens fiber differentiation [24]. In addition, β1, α6A, and α6B mRNAs and proteins are distinctly localized along basolateral surfaces of rat lens fibers, particularly during early fiber differentiation below the lens equator and at the posterior pole [24]. This indicates the involvement of α6Aβ1 and/or α6Bβ1 integrins in cell–cell interactions, particularly attachment and migration of the apical tips of elongating fibers along the epithelial–fiber interface and fiber–fiber cell interactions.

The studies of other stem cell populations support the ratio of α6 cytoplasmic variants as an important indicator of whether stem cells remain undifferentiated or undergo differentiation. It has been reported that α6B is predominantly expressed in undifferentiated visceral endoderm, parietal endoderm, and embryonic stem cells. When these cells are induced to differentiate, α6A expression is upregulated and predominates over α6B [25–27]. In embryonic mouse kidney, α6B is the major splice variant and α6Bβ1 plays a role in the conversion of nephrogenic mesenchyme to epithelial tubules [28]. Fetal testis exclusively expresses α6B, whereas both α6A(β1) and α6B(β1) are expressed when differentiation is induced in pre-pubertal testes and Sertoli-spermatogenic cell co-cultures [29]. Taken together, these findings strongly suggest that the distinct α6 cytoplasmic variants have differential functions in the developing embryos.

There is evidence that α6 cytoplasmic variants contribute to adult stem/progenitor cell properties as well. Breast cancer stem cells (CSCs), characterized by CD44^high^/CD24^low^, consist of epithelial and mesenchymal cells. The epithelial population predominantly expresses α6A(β1), whereas the mesenchymal population predominantly expresses α6B(β1) [12]. The function of breast CSCs appears to depend on the relative expression of α6B(β1). α6A(β1) expression is not required for breast CSC properties [12], and this study suggests that these are manifested primarily by the mesenchymal cell population which is characterized by a high ratio of α6B/α6A. In contrast to breast CSCs, undifferentiated human intestinal cells predominantly express α6A(β4), whereas α6B(β4) expression is mainly detected in differentiated cells. This finding suggests that a high α6B/α6A ratio is permissive for enterocytic differentiation [30].

### α6 Cytoplasmic variants in stem cell fate decision

Integrin-mediated cell–extracellular matrix (ECM) interactions transmit biochemical and mechanical signals from the ECM to the interior of cells via the cytoplasmic domains of integrins [31, 32]. The cytoplasmic domains of integrins typically interact with cytosolic adaptor proteins and/or kinases to further activate the downstream signals. In embryonic lens cells, α6A interacts with adaptor protein Shc and the downstream effector Grb2 to form a complex [18]. The greatest amount of the complex is found in the differentiating cortical fiber cell zone. α6A in the cortical fiber region interacts with the cytoskeleton and is associated with activation of specific cell signals for lens cell differentiation [18]. An additional study has shown that α6A(β1 and/or β4) expression is required for acquisition of a migratory cell phenotype accompanying lens cell differentiation [23].

The cytoplasmic domain of α6A, specifically the 11 amino acids at the C-terminus of the α6A cytoplasmic tail, inhibits proliferation and promotes terminal differentiation of primary quail myoblasts by suppression of focal adhesion kinase (FAK) and mitogen-activated protein kinase (MAPK) [33]. The findings suggest that α6A cytoplasmic domain-dependent changes in focal adhesion signals regulate the withdrawal of myoblasts from the cell cycle and initiation of terminal differentiation. GATA-4 is known to be a critical transcription factor in the development of cardiac muscles [34]. Temporal correlation of α6A, GATA-4, and myosin light chain-2 V (a cardiac-muscle-specific marker) has been observed during mouse embryonic stem cell differentiation [35], suggesting that α6A(β1) may be involved in activation of GATA-4 signals which in turn direct the development of cardiac muscles.

**Fig. 2:**
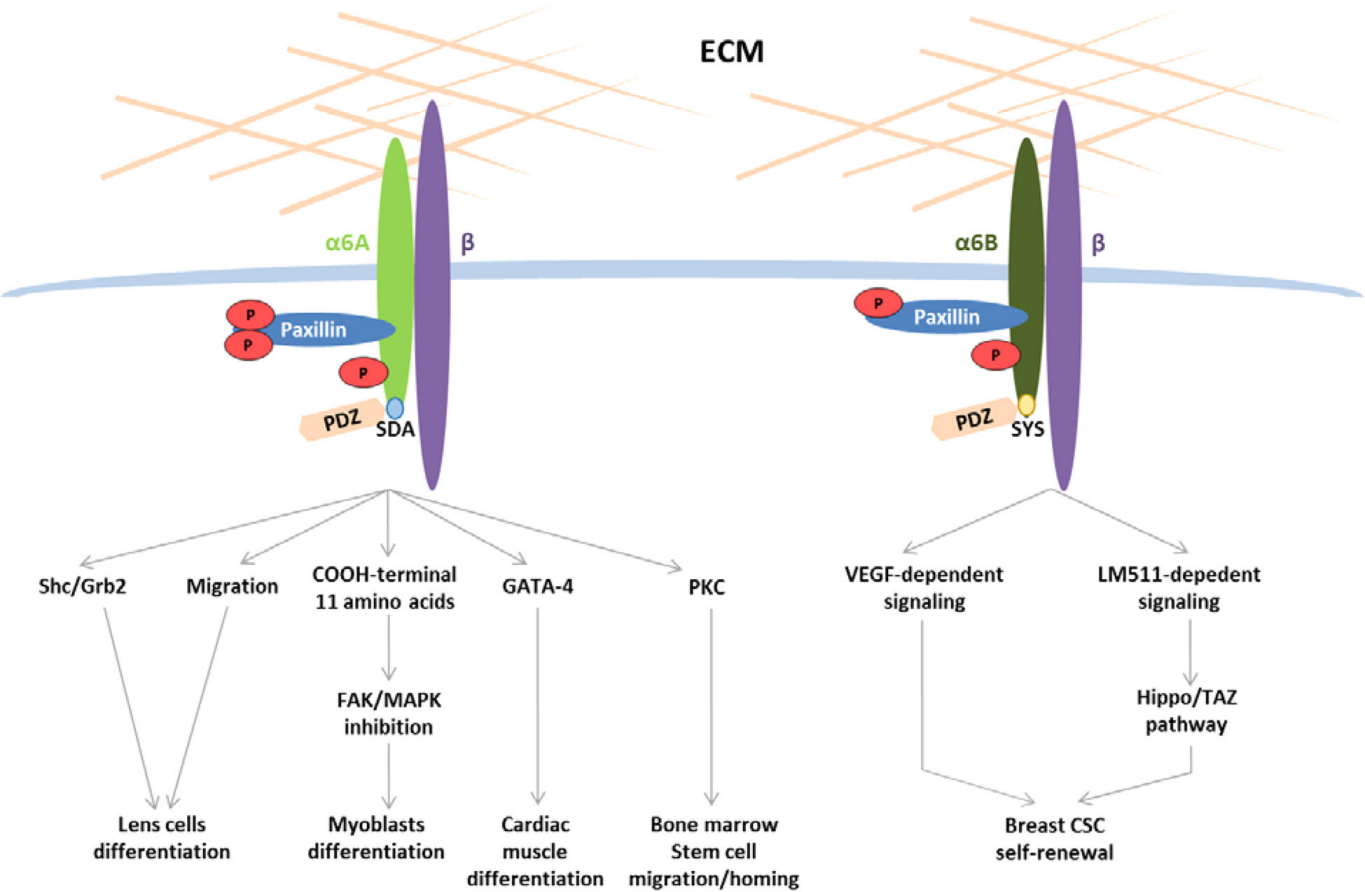
Summary of the potential signal pathways involved in regulation of stem cell fate by distinct α6 cytoplasmic variants. Current findings suggest that α6A functions to promote stem cell differentiation in various stem cell populations via specific signal pathways. α6B mediates VEGF- and/or LM511-dependent signals to promote the self-renewal of breast CSCs

The cytoplasmic domains of both α6A and α6B contain serine, threonine, and tyrosine residues which could serve as potential phosphorylation sites. In fact, it has been shown that macrophage adhesion to laminin substrates promotes α6 phosphorylation in the cytoplasmic domain [36]. In addition to phosphorylation of their own residues, the cytoplasmic domains of α6A and α6B also mediate differential tyrosine phosphorylation of paxillin and other signaling molecules [37]. It is predicted that altered phosphorylation of α6A and α6B cytoplasmic portions and/or α6A/B-mediated differential phosphorylation of downstream signal molecules may activate distinct intracellular signals that could potentially regulate the stem cell fate propensity. PDZ domain, a structural fold in many signal molecules, recognizes the C-terminus of membrane-anchored proteins, including integrins [38]. The last three amino acids (SDA) in the cytoplasmic tail of α6A are a typical PDZ-binding motif, whereas the corresponding amino acids (SYS) in the cytoplasmic tail of α6B represent an alternative PDZ-binding motif. It has been reported that the SYS motif in α6B is less efficient in binding to the signal molecules than the SDA motif in α6A [39]. Thus, altered PDZ-binding motifs in the cytoplasmic domains of α6A versus α6B might mechanistically link to the stem cell fate determination. Collectively, alterative splicing of the α6 cytoplasmic domain may mediate differential intracellular signals that direct stem cell fate decision (Fig. 2).

### α6-Integrin in stem cell–niche interaction

Stem cells reside in a unique microenvironment known as the stem cell “niche”. The stem cell niche typically constitutes stem cells, support cells, and the ECM [40]. The niche supports and instructs establishment and maintenance of the stem cell population [41]. Integrins, as a microenvironmental sensor, mediate interactions between stem/support cells and the surrounding ECM. The cell-ECM interactions are critical for niche morphogenesis, anchorage of stem and support cells, positioning of dividing stem cells, and controlling the balance between stem cell renewal and differentiation [42].

The ability of stem cells to reside in the niche for a long period of time is paramount for tissue homeostasis and regeneration. α6-Integrin-mediated ECM adhesion is critical for anchorage and long-term maintenance of a variety of stem cell populations in the niche. Disruption of interactions between α6β1 and laminin in the lateral ventricle of mice by a function-blocking anti-α6 antibody causes the release of NSCs in the subventricular zone and activation of NSCs [43]. Studies also support that α6(β1) integrins play an essential role in anchoring spermatogonial stem cells [44] and hematopoietic stem cells [45], and α6(β4) in anchoring dermal stem cells [46] to their niches.

Mammary gland stem cells and interfollicular epidermal stem cells express higher levels of α6 and β1 integrin subunits [47, 48]. In breast cancer, α6(β1) expression promotes self-renewal of tumor-initiating cells (TICs) and mediates transduction of cell signals essential for establishment of an autocrine loop to maintain the TIC niche [49, 50]. Ablation of α6β1 results in random orientation of the basal cell division plane and impairs epithelial homeostasis [51]. Furthermore, laminin511 (LM511) engagement of α6β1 supports the self-renewal of mouse ESCs, whereas internalization of α6β1 promotes mESC differentiation towards an epithelial lineage via a FAK/Akt/Erk-dependent mechanism [52]. In contrast, LM532–α6 interactions direct differentiation of ventral ectodermal ridge (VER) progenitor cells in developing mouse tails [53]. It remains to be determined whether mESC and VER progenitor cells express distinct α6 cytoplasmic variants and whether this is responsible for the differential responses (self-renewal vs differentiation) of these two stem cell populations to laminin engagement of α6.

Currently, there is limited information for the role of α6 cytoplasmic variants in the regulation of stem cell–niche interactions. CD34^+^ and CD34^+^CD38^−^ bone marrow stem/progenitor cells express both α6A and α6B [54]. Although α6A and α6B are equally associated with the β1 subunit and also have similar specificity and affinity for ligand binding, it was found that α6A(β1), but not α6B(β1), was responsible for protein kinase C–dependent activation of MAP kinases. α6A(β1) was also found to be more active than α6B(β1) in promoting migration of bone marrow stem cells [54]. Together, these data suggest that α6A may be important in migration and mobilization of hematopoietic stem/progenitor cells in the bone marrow stem cell niche during hematopoiesis. In addition, breast CSCs express a high level of LM511, which promotes the formation of a LM511 matrix niche critical for breast CSC self-renewal and tumor initiation [55]. Laminin engagement of α6B(β1) activates the Hippo transducer TAZ, which upregulates the transcription of the α5 subunit in LM511. The latter finding suggests that α6B(β1) integrins mediate the establishment of a positive feedback loop between breast CSCs and the niche, which functions to maintain breast CSC self-renewal and cancer formation.

## Conclusions

The α6-integrin subunit is a common stem cell marker in diverse tissues. As a matrix adhesion molecule, α6-integrins play important functional roles in the anchorage of stem cells within the niche, maintenance of stem cell stemness, regulation of stem cell differentiation, orientation of dividing stem cells, and migration and/or homing of hematopoietic stem/progenitor cells to the niche of bone marrow. Alternative splicing impacts the function of α6 in the regulation of the stem cell propensity and niche interaction. The spatial and temporal expression of distinct α6 cytoplasmic variants in both embryonic and adult stem cells suggests that α6A and α6B have distinct functions during embryogenesis and at the adult age. The cytoplasmic variants of the α6 subunit provide an excellent platform for the study of cell signals important for stem cell self-renewal and differentiation, while deregulation of these signals may underlie a wide range of human diseases. We believe that dissecting the multifaceted functions of α6 splice variants would inform the rational design of novel stem cell-based therapies for a range of human diseases.

## Abbreviations

CSC: Cancer stem cell
ECM: Extracellular matrix
ESCs: Embryonic stem cells
ESPR1: Epithelial splicing regulatory protein 1
FAK: Focal adhesion kinase
HSCs: Hematopoietic stem cells
LM: Laminin
MAPK: Mitogen-activated protein kinase
NCBI: National Center for Biotechnology Information
NSCs: Neural stem cells
ORF: Open reading frame
TICs: Tumor-initiating cells
VER: Ventral ectodermal ridge

## References

[1] Sonnenberg A, Modderman PW, Hogervorst F (1988). Laminin receptor on platelets is the integrin VLA-6. Nature.

[2] Stepp MA, Spurr-Michaud S, Tisdale A, Elwell J, Gipson IK (1990). Alpha 6 beta 4 integrin heterodimer is a component of hemidesmosomes. Proc Natl Acad Sci U S A.

[3] Hemler ME, Crouse C, Sonnenberg A (1989). Association of the VLA alpha 6 subunit with a novel protein. A possible alternative to the common VLA beta 1 subunit on certain cell lines. J Biol Chem.

[4] Tamura RN, Rozzo C, Starr L, Chambers J, Reichardt LF, Cooper HM, Quaranta V (1990). Epithelial integrin alpha 6 beta 4: complete primary structure of alpha 6 and variant forms of beta 4. J Cell Biol.

[5] Krebsbach PH, Villa-Diaz LG (2017). The role of integrin α6 (CD49f) in stem cells: more than a conserved biomarker. Stem Cells Dev.

[6] Ramalho-Santos M, Yoon S, Matsuzaki Y, Mulligan RC, Melton DA (2002). “Stemness”: transcriptional profiling of embryonic and adult stem cells. Science.

[7] Ivanova NB, Dimos JT, Schaniel C, Hackney JA, Moore KA, Lemischka IR (2002). A stem cell molecular signature. Science.

[8] Fortunel NO, Otu HH, Ng HH, Chen J, Mu X, Chevassut T, Li X, Joseph M, Bailey C, Hatzfeld JA, Hatzfeld A, Usta F, Vega VB, Long PM, Libermann TA, Lim B (2003). Comment on “ ‘Stemness’: transcriptional profiling of embryonic and adult stem cells” and “a stem cell molecular signature”. Science.

[9] Hogervorst F, Kuikman I, van Kessel AG, Sonnenberg A (1991). Molecular cloning of the human alpha 6 integrin subunit. Alternative splicing of alpha 6 mRNA and chromosomal localization of the alpha 6 and beta 4 genes. Eur J Biochem.

[10] Tamura RN, Cooper HM, Collo G, Quaranta V (1991). Cell type-specific integrin variants with alternative alpha chain cytoplasmic domains. Proc Natl Acad Sci U S A.

[11] Warzecha CC, Jiang P, Amirikian K, Dittmar KA, Lu H, Shen S, Guo W, Xing Y, Carstens RP (2010). An ESRP-regulated splicing programme is abrogated during the epithelial-mesenchymal transition. EMBO J.

[12] Goel HL, Gritsko T, Pursell B, Chang C, Shultz LD, Greiner DL, Norum JH, Toftgard R, Shaw LM, Mercurio AM (2014). Regulated splicing of the α6 integrin cytoplasmic domain determines the fate of breast cancer stem cells. Cell Rep.

[13] Ziober BL, Vu MP, Waleh N, Crawford J, Lin CS, Kramer RH (1993). Alternative extracellular and cytoplasmic domains of the integrin alpha 7 subunit are differentially expressed during development. J Biol Chem.

[14] Delwel GO, Kuikman I, Sonnenberg A (1995). An alternatively spliced exon in the extracellular domain of the human alpha 6 integrin subunit--functional analysis of the alpha 6 integrin variants. Cell Adhes Commun.

[15] Davis TL, Rabinovitz I, Futscher BW, Schnölzer M, Burger F, Liu Y, Kulesz-Martin M, Cress AE (2001). Identification of a novel structural variant of the alpha 6 integrin. J Biol Chem.

[16] Demetriou MC, Pennington ME, Nagle RB, Cress AE (2004). Extracellular alpha 6 integrin cleavage by urokinase-type plasminogen activator in human prostate cancer. Exp Cell Res.

[17] Lehmann M, Rigot V, Seidah NG, Marvaldi J, Lissitzky JC (1996). Lack of integrin alpha-chain endoproteolytic cleavage in furin-deficient human colon adenocarcinoma cells LoVo. Biochem J.

[18] Walker JL, Zhang L, Menko AS (2002). A signaling role for the uncleaved form of alpha 6 integrin in differentiating lens fiber cells. Dev Biol.

[19] Delwel GO, Hogervorst F, Sonnenberg A (1996). Cleavage of the alpha6A subunit is essential for activation of the alpha6Abeta1 integrin by phorbol 12-myristate 13-acetate. J Biol Chem.

[20] Thorsteinsdóttir S, Roelen BA, Freund E, Gaspar AC, Sonnenberg A, Mummery CL (1995). Expression patterns of laminin receptor splice variants alpha 6A beta 1 and alpha 6B beta 1 suggest different roles in mouse development. Dev Dyn.

[21] Collo G, Domanico SZ, Klier G, Quaranta V (1995). Gradient of integrin alpha 6A distribution in the myocardium during early heart development. Cell Adhes Commun.

[22] Gimond C, Baudoin C, van der Neut R, Kramer D, Calafat J, Sonnenberg A (1998). Cre-loxP-mediated inactivation of the alpha6A integrin splice variant in vivo: evidence for a specific functional role of alpha6A in lymphocyte migration but not in heart development. J Cell Biol.

[23] Walker JL, Menko AS (1999). alpha6 Integrin is regulated with lens cell differentiation by linkage to the cytoskeleton and isoform switching. Dev Biol.

[24] Wederell ED, Brown H, O'connor M, Chamberlain CG, McAvoy JW, de Iongh RU (2005). Laminin-binding integrins in rat lens morphogenesis and their regulation during fibre differentiation. Exp Eye Res.

[25] Cooper HM, Tamura RN, Quaranta V (1991). The major laminin receptor of mouse embryonic stem cells is a novel isoform of the alpha 6 beta 1 integrin. J Cell Biol.

[26] Jiang R, Grabel LB (1995). Function and differential regulation of the alpha 6 integrin isoforms during parietal endoderm differentiation. Exp Cell Res.

[27] Morini M, Piccini D, De Santanna A, Levi G, Barbieri O, Astigiano S (1999). Localization and expression of integrin subunits in the embryoid bodies of F9 teratocarcinoma cells. Exp Cell Res.

[28] Falk M, Salmivirta K, Durbeej M, Larsson E, Ekblom M, Vestweber D, Ekblom P (1996). Integrin alpha 6B beta 1 is involved in kidney tubulogenesis in vitro. J Cell Sci.

[29] Kierszenbaum AL, Rosselot C, Rivkin E, Tres LL (2006). Role of integrins, tetraspanins, and ADAM proteins during the development of apoptotic bodies by spermatogenic cells. Mol Reprod Dev.

[30] Dydensborg AB, Teller IC, Basora N, Groulx JF, Auclair J, Francoeur C, Escaffit F, Paré F, Herring E, Ménard D, Beaulieu JF (2009). Differential expression of the integrins alpha6Abeta4 and alpha6Bbeta4 along the crypt-villus axis in the human small intestine. Histochem Cell Biol.

[31] Sastry SK, Horwitz AF (1993). Integrin cytoplasmic domains: mediators of cytoskeletal linkages and extra- and intracellular initiated transmembrane signaling. Curr Opin Cell Biol.

[32] Chen H, Qu J, Huang X, Kurundkar A, Zhu L, Yang N, Venado A, Ding Q, Liu G, Antony VB, Thannickal VJ, Zhou Y (2016). Mechanosensing by the α6-integrin confers an invasive fibroblast phenotype and mediates lung fibrosis. Nat Commun.

[33] Sastry SK, Lakonishok M, Wu S, Truong TQ, Huttenlocher A, Turner CE, Horwitz AF (1999). Quantitative changes in integrin and focal adhesion signaling regulate myoblast cell cycle withdrawal. J Cell Biol.

[34] Grépin C, Nemer G, Nemer M (1997). Enhanced cardiogenesis in embryonic stem cells overexpressing the GATA-4 transcription factor. Development.

[35] Thorsteinsdóttir S, Roelen BA, Goumans MJ, Ward-van Oostwaard D, Gaspar AC, Mummery CL (1999). Expression of the alpha 6A integrin splice variant in developing mouse embryonic stem cell aggregates and correlation with cardiac muscle differentiation. Differentiation.

[36] Shaw LM, Messier JM, Mercurio AM (1990). The activation dependent adhesion of macrophages to laminin involves cytoskeletal anchoring and phosphorylation of the alpha 6 beta 1 integrin. J Cell Biol.

[37] Shaw LM, Turner CE, Mercurio AM (1995). The alpha 6A beta 1 and alpha 6B beta 1 integrin variants signal differences in the tyrosine phosphorylation of paxillin and other proteins. J Biol Chem.

[38] Fanning AS, Anderson JM (1999). PDZ domains: fundamental building blocks in the organization of protein complexes at the plasma membrane. J Clin Invest.

[39] El Mourabit H, Poinat P, Koster J, Sondermann H, Wixler V, Wegener E, Laplantine E, Geerts D, Georges-Labouesse E, Sonnenberg A, Aumailley M (2002). The PDZ domain of TIP-2/GIPC interacts with the C-terminus of the integrin alpha5 and alpha6 subunits. Matrix Biol.

[40] Raymond K, Deugnier MA, Faraldo MM, Glukhova MA (2009). Adhesion within the stem cell niches. Curr Opin Cell Biol.

[41] Gönczy P, Viswanathan S, DiNardo S (1992). Probing spermatogenesis in Drosophila with P-element enhancer detectors. Development.

[42] Ellis SJ, Tanentzapf G (2010). Integrin-mediated adhesion and stem-cell-niche interactions. Cell Tissue Res.

[43] Shen Q, Wang Y, Kokovay E, Lin G, Chuang SM, Goderie SK, Roysam B, Temple S (2008). Adult SVZ stem cells lie in a vascular niche: a quantitative analysis of niche cell-cell interactions. Cell Stem Cell.

[44] Shinohara T, Avarbock MR, Brinster RL (1999). beta1- and alpha6-integrin are surface markers on mouse spermatogonial stem cells. Proc Natl Acad Sci U S A.

[45] Qian H, Tryggvason K, Jacobsen SE, Ekblom M (2006). Contribution of alpha6 integrins to hematopoietic stem and progenitor cell homing to bone marrow and collaboration with alpha4 integrins. Blood.

[46] Watt FM (2002). Role of integrins in regulating epidermal adhesion, growth and differentiation. EMBO J.

[47] Stingl J, Eirew P, Ricketson I, Shackleton M, Vaillant F, Choi D, Li HI, Eaves CJ (2006). Purification and unique properties of mammary epithelial stem cells. Nature.

[48] Jones PH, Simons BD, Watt FM (2007). Sic transit gloria: farewell to the epidermal transit amplifying cell?. Cell Stem Cell.

[49] Lo PK, Kanojia D, Liu X, Singh UP, Berger FG, Wang Q, Chen H (2012). CD49f and CD61 identify Her2/neu-induced mammary tumor-initiating cells that are potentially derived from luminal progenitors and maintained by the integrin-TGFβ signaling. Oncogene.

[50] Goel HL, Pursell B, Chang C, Shaw LM, Mao J, Simin K, Kumar P, Vander Kooi CW, Shultz LD, Greiner DL, Norum JH, Toftgard R, Kuperwasser C, Mercurio AM (2013). GLI1 regulates a novel neuropilin-2/α6β1 integrin based autocrine pathway that contributes to breast cancer initiation. EMBO Mol Med.

[51] Akhtar N, Streuli CH (2013). An integrin-ILK-microtubule network orients cell polarity and lumen formation in glandular epithelium. Nat Cell Biol.

[52] Domogatskaya A, Rodin S, Boutaud A, Tryggvason K (2008). Laminin-511 but not −332, −111, or −411 enables mouse embryonic stem cell self-renewal in vitro. Stem Cells.

[53] Lopez-Escobar B, De Felipe B, Sanchez-Alcazar JA, Sasaki T, Copp AJ, Ybot-Gonzalez P (2012). Laminin and integrin expression in the ventral ectodermal ridge of the mouse embryo: implications for regulation of BMP signalling. Dev Dyn.

[54] Gu YC, Kortesmaa J, Tryggvason K, Persson J, Ekblom P, Jacobsen SE, Ekblom M (2003). Laminin isoform-specific promotion of adhesion and migration of human bone marrow progenitor cells. Blood.

[55] Chang C, Goel HL, Gao H, Pursell B, Shultz LD, Greiner DL, Ingerpuu S, Patarroyo M, Cao S, Lim E, Mao J, McKee KK, Yurchenco PD, Mercurio AM (2015). A laminin 511 matrix is regulated by TAZ and functions as the ligand for the α6Bβ1 integrin to sustain breast cancer stem cells. Genes Dev.

